# A Systematic Review of the Different Calculation Methods for Measuring Jump Height During the Countermovement and Drop Jump Tests

**DOI:** 10.1007/s40279-023-01828-x

**Published:** 2023-03-20

**Authors:** Jiaqing Xu, Anthony Turner, Paul Comfort, John R. Harry, John J. McMahon, Shyam Chavda, Chris Bishop

**Affiliations:** 1 Faculty of Science and Technology, London Sport Institute, Middlesex University, London, UK; 2 Directorate of Psychology and Sport, University of Salford, Salford, UK; 3 Human Performance & Biomechanics Laboratory, Department of Kinesiology & Sport Management, Texas Tech University, Lubbock, TX USA

## Abstract

**Background:**

The heights obtained during the countermovement jump and drop jump tests have been measured by numerous studies using different calculation methods and pieces of equipment. However, the differences in calculation methods and equipment used have resulted in discrepancies in jump height being reported.

**Objectives:**

The aim of this systematic review was to examine the available literature pertaining to the different calculation methods to estimate the jump height during the countermovement jump and drop jump.

**Methods:**

A systematic review of the literature was undertaken using the SPORTDiscus, MEDLINE, CINAHL, and PubMed electronic databases, with all articles required to meet specified criteria based on a quality scoring system.

**Results:**

Twenty-one articles met the inclusion criteria, relating various calculation methods and equipment employed when measuring jump height in either of these two tests. The flight time and jump-and-reach methods provide practitioners with jump height data in the shortest time, but their accuracy is affected by factors such as participant conditions or equipment sensitivity. The motion capture systems and the double integration method measure the jump height from the centre of mass height at the initial flat foot standing to the apex of jumping, where the centre of mass displacement generated by the ankle plantarflexion is known. The impulse-momentum and flight time methods could only measure the jump height from the centre of mass height at the instant of take-off to the apex of jumping, thus, providing statistically significantly lower jump height values compared with the former two methods. However, further research is warranted to investigate the reliability of each calculation method when using different equipment settings.

**Conclusions:**

Our findings indicate that using the impulse-momentum method via a force platform is the most appropriate way for the jump height from the instant of take-off to the apex of jumping to be measured. Alternatively, the double integration method via a force platform is preferred to quantify the jump height from the initial flat foot standing to the apex of jumping.

## Key Points

**Table:** 

There are currently five different calculation methods to measure jump height during the countermovement and drop jump tests. However, each method has its own set of limitations due to factors such as: equipment selection, participant condition or the calculation process.
The impulse-momentum method (via a force platform) is more reliable to quantify the jump height from the centre of mass height at the take-off instant to the apex of the jump during both countermovement and drop jump actions. This method removes many confounding variables when using the flight time method, such as the asymmetric take-off and landing position.
The double integration method (via a force platform) provides reliable jump height from the centre of mass height at the normal standing to the apex of the jump. The double integration method requires less time on data processing and equipment preparation compared to motion capture systems.

## Introduction

Jumping is commonly performed during competitive sports, which is an action requiring the coordination of multiple joints and muscles [1, 2]. During vertical jumping, a main objective is to leave the ground and move the body’s centre of mass (COM) upwards as high as possible, whereby jump performance is reflected by the value of the jump height (JH) [2]. Typically, JH is defined as the COM displacement between the height of the COM during normal standing and the peak COM height (i.e. apex) of the jump (denoted as JH-1 in this article) [3, 4]. Alternatively, JH can also be defined as the COM displacement between COM height at the take-off instant and the apex of the jump (denoted as JH-2 in this article, and can also be referred to as flight distance) [2]. Noting that both JH-1 and JH-2 are commonly applied to evaluate JH, it is important to appreciate that their definitions and how they are determined are different [1, 5–9]. Specifically, the JH-1 considers the work of ankle plantarflexion and the rise of the COM position before the take-off instant, whereas, the JH-2 ignores the take-off COM height into its calculation and measures the flight distance, which is only one component of JH-1 [1, 2, 10]. Whilst numerous jump types exist, two of the most commonly used in practice are the countermovement jump (CMJ) and the drop jump (DJ). The CMJ is a simple and practical test to measure an athlete’s lower body impulse capacity or rather, ‘ballistic force-production capability’ [6], particularly when athletes are required to jump as high as possible [7, 8]. Thus, it is suggested that practitioners measure metrics such as countermovement depth, time to take-off, JH and reactive strength index modified (i.e., a ratio between JH and time to take-off) to provide an understanding of both CMJ outcome measures and the jump strategy utilised [11]. When considering the DJ, this test starts by stepping off a box at a fixed height [12, 13], landing on the floor and rebounding immediately in the vertical direction with the intention of minimising ground contact time and maximising JH [14, 15]. The DJ is used to evaluate whether athletes can rapidly perform the stretch–shortening cycle (SSC) [14]. This ability is typically reflected in the metric referred to as a reactive strength index, which is calculated as JH divided by ground contact time [14]. Given the CMJ represents a long SSC action (SSC duration ≥ 250 ms) and the DJ represents a short SSC action (SSC duration ≤ 250 ms) [16, 17], it is likely that monitoring JH during these two jump actions is warranted to provide a holistic evaluation of an athletes’ jump performance [17–19].

There are numerous pieces of equipment available to measure parameters required for JH calculations during both of these jump tasks. For example, force–time data is recorded by force platforms (FP), or the position-time data is recorded by three-dimensional (3D) motion capture systems [2]. Subsequently, JH is obtained through vertical ground reaction force (vGRF) analyses and displacement calculations from reflective marker positions, respectively [15, 20, 21]. In contrast, the FP or 3D motion capture technologies may not always be favourable when budgets are finite, thus a linear position transducer may provide a cheaper and more viable choice of equipment, when aiming to measure JH [22, 23]. In addition, some FP or 3D cameras are not transportable and therefore, practitioners typically use jump mats [15, 24, 25], simplified optical measurement systems (e.g. photocell mat or laser beam) [21, 25–27] or smartphone applications [28] to record the flight time (FT) for JH-2 calculations. Practitioners also use hardware-only vertical jump systems (e.g. Vertec vanes jump device or Sargent jump) to measure the ‘jump-and-reach’ height [29, 30]. Whilst other practitioners select accelerometers to acquire peak velocity which occurs just prior to take-off (i.e. the instant that the vertical COM displacement achieves zero) during CMJ or the touchdown velocity during DJ [18, 27, 31]. Among the aforementioned equipment, the FP and 3D motion capture systems are considered the gold standard given their accuracy for calculating JH and all associated kinetic and kinematics variables [21, 30, 32]. However, each piece of equipment has its strengths and weaknesses. For example, some FP cannot provide the measured outcomes instantly, where the treatment of vGRF data requires time and specific data analytical skills [33]. In addition, the motion capture systems require rather extensive set-up processes (e.g. calibration, precise marker attachment and data processing in specific software) [15, 27]. Consequently, these characteristics largely prevent practitioners from using such systems when working in the field [21, 27], resulting in the use of smartphone applications or jump mats, which provide JH-2 values instantaneously. However, the calculation method for these pieces of equipment is restricted to the imprecise FT method, owing to the lack of vGRF data [29]. Ultimately, the technology and calculation method(s) used to report JH can compromise the validity, reliability and accuracy of the data, which collectively determine its utility in practice [21].

A number of different methods are available to calculate JH [1, 29, 32, 34]. These methods can be divided into two “groups” according to how rapidly or user free the outcome is provided to practitioners, i.e. indirect, and direct methods [32]. The indirect methods include the FT method, the impulse-momentum (IM) method and the double integration method, where these methods involve several mathematical calculation processes and potential errors in their calculations. When applying indirect methods, JH is calculated based on the COM kinematics and kinetic parameters, such as the FT and vGRF provided by the FP or accelerometer [1, 2, 32]. In the direct methods, the JH-1 is directly provided by the vertical jump systems [20, 30, 31] or is acquired by the position-time data resulting from the motion capture systems (i.e. including 3D motion capture systems or a two-dimensional [high-speed] video camera) [15, 35, 36]. However, at present, the recommendations for calculation methods are somewhat inconsistent among existing studies. Noting that even when using the same equipment, all methods also have both technology and user-generated limitations [2]. These apparent discrepancies provide important considerations for practitioners regarding the process by which we administer jump testing, the equipment we use and the calculation methods employed to derive the outcome measure. Therefore, it is important to understand how to accurately measure the JH during the CMJ and DJ under different experimental designs.

The primary aim of this systematic review was to examine the available literature pertaining to the different calculation methods to estimate JH during the CMJ and DJ tests. More specifically, we sought to critically evaluate the reliability, equipment selections, and the strengths and weaknesses of each method.

## Methodology

### Study Design

This systematic review was conducted under the Preferred Reporting Items for Systematic Reviews and Meta-Analysis (PRISMA) statement in 2020 [37]. A review protocol was not pre-registered for this review.

### Literature Search Methodology

**Fig. 1:**
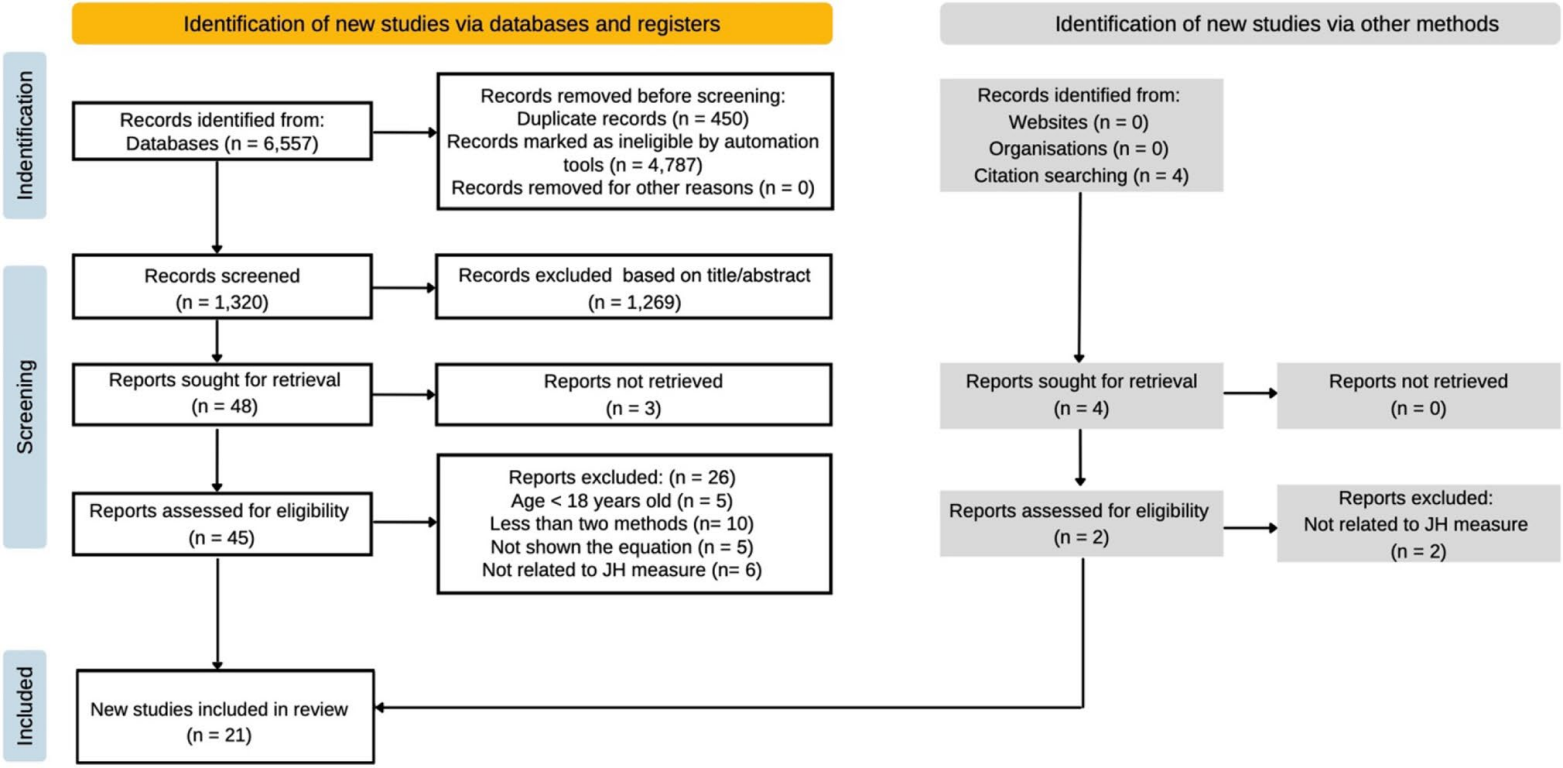
Flow diagram illustrating the identification and selection of studies for the current review. *JH* jump height

Original and review journal articles were searched from SPORTDiscus, MEDLINE, CINAHL and PubMed electronic databases (publication date from 2000 to 2022). Figure 1 provides a schematic outline of the search methodology. The search strategy combined three main terms as: “Jump”, “Method*” and “Jump Height*”, where these terms were used and combined under Boolean’s language with the operators AND and OR. Term 1: Countermovement, countermovement vertical, counter-movement, CMJ, Drop, DJ. Term 2: Calculat*, Measur*, Estimat*. Term 3: Vertical displacement, Cent* of mass vertical, COM, Flight. If full-text articles were not available in the aforementioned electronic databases, then further searches were conducted in Google Scholar and ResearchGate™ websites. Additional studies were identified by reading through the reference lists of the database searched studies. The final search date for literature was 20 January, 2022.

### Inclusion Criteria

Studies were included if they met the following criteria: (1) implemented at least two or more calculation methods or pieces of equipment in the outcome measure; (2) clearly described equations of each calculation method, equipment information (i.e. type and sampling frequency) and jump actions (i.e. CMJ or DJ); included healthy adult participants (i.e. aged ≥ 18 years old); (3) presented full data (mean and standard deviation) and statistical significance in results; (4) the drafts were written in English and were published in a peer-reviewed journal. For the purpose of this systematic review, the included articles were required to describe methods used to measure JH during CMJ and/or DJ. As such, articles that simply measured JH in their experimental designs were excluded.

### Grading Article Quality

The quality scoring system used in the present study was adapted and modified from Bishop et al. [38]. Each study was appraised using eight criteria (see Table 1) and a scale of 0–2 (i.e. zero = no, one = maybe, and two = yes). As none of the JH measurement studies included in this systematic review had training interventions, the sixth criteria pertaining to “Training duration practical” was removed from the scale, leaving eight criteria yielding a maximum of 16 points. The total scores of each study were then converted to a percentage ranging from 0 to 100%. To ensure that the article quality assessment was equitable, only articles that scored > 75% were included in the final analysis [38], as shown in Table 2.

**Table 1:** Study quality scoring system (adapted from Bishop et al. [38])

Criteria no	Item	Score
1	Inclusion criteria stated	0–2
2	Subjects assigned appropriately	0–2
3	Procedures described (equations, equipment setting, jump actions)	0–2
4	Dependent variables defined^a^	0–2
5	Assessments practical (easy to implement)	0–2
6	Statistics appropriate (reliability, significant differences)	0–2
7	Results detailed (mean, standard deviation)	0–2
8	Conclusions insightful (clear, practical application, future directions)	0–2
Total		0–16

^a^The fourth item includes the definition of first meaningful change in vertical ground reaction force on the force–time curve, the instant of countermovement and drop action, instant of take-off and landing

**Table 2:** Results of study quality scoring

References	Criteria no								
	1	2	3	4	5	6	7	8	Total, *n* (%)
Reeve and Tyler [24]	2	1	2	2	2	1	2	2	14 (87.50%)
Moir [5]	2	2	2	2	2	2	2	2	16 (100.00%)
Wade et al. [2]	2	1	2	2	2	1	2	2	14 (87.50%)
Moir et al. [39]	2	2	2	2	2	2	2	2	16 (100.00%)
Pérez-Castilla et al. [42]	2	1	2	2	2	2	2	2	15 (93.75%)
Dias et al. [15]	2	2	2	0	2	1	2	2	13 (81.25%)
Martínez-Martí et al. [26]	2	1	2	2	2	1	2	1	13 (81.25%)
Buckthorpe et al. [41]	2	2	2	2	2	1	2	2	15 (93.75%)
Whitmer et al. [40]	2	2	2	1	2	1	2	2	14 (87.50%)
García-López et al. [45]	2	2	2	2	2	2	2	2	16 (100.00%)
Heredia-Jimenez and Orantes-Gonzalez [27]	2	1	2	0	2	2	2	2	13 (81.25%)
Słomka et al. [21]	2	2	2	2	2	2	2	2	16 (100.00%)
Bui et al. [30]	2	2	1	1	2	1	2	2	13 (81.25%)
Leard et al. [29]	2	2	1	1	2	1	2	2	13 (81.25%)
Nuzzo et al. [20]	2	2	1	2	2	2	2	2	15 (93.75%)
Aragón [3]	2	1	2	2	2	2	2	2	15 (93.75%)
Conceição et al. [32]	2	2	2	2	2	0	2	2	14 (87.50%)
Chiu and Dæhlin [1]	2	2	2	2	2	0	2	2	14 (87.50%)
Wank and Coenning [36]	2	1	2	2	2	1	2	2	14 (87.50%)
Baca [35]	2	1	2	1	2	1	2	2	13 (81.25%)
Brooks et al. [28]	2	2	2	0	2	2	2	2	14 (87.50%)

## Results

### Literature Search Results

A total of 6557 articles were initially returned with an additional four articles included from other sources [1, 32, 35, 36]. After excluding 5237 duplicates and articles not published in sport-related journals, 1320 articles were selected to be screened by title and abstract, followed by 48 articles being read to ensure that they were related to the inclusion criteria. According to the quality score system and the eligibility of the full text of these articles, 21 articles scored > 75% and were included in the systematic review. Figure 1 illustrates the search strategy [37]. The assessment of the study quality is reported in Table 2, where the mean quality score was 88% (range 81–100%). The characteristic of the 21 included studies is shown in Table 3.

**Table 3:** Characteristics of studies included in this systematic review

Reference	Subjects	Jump actions	Calculation method	Equipment	Reliability/variability
Reeve and Tyler [24]	15 male; 8 female	3 CMJ^a^; 3 CMJ^b^	IM; FT	FP (1202 Hz)^c^ Jump mat (1000 Hz)	Not provided
Moir [5]	50 male; 50 female	3 CMJ^b^	IM; FT; IM + S (by DI)	FP (1202 Hz)	A high degree of consistency across methods in male participants (ICC = 0.927; 95% CI 0.887–0.955); in female participants (ICC = 0.934; 95% CI 0.897–0.960)
Wade et al. [2]	15 male; 9 female	5 CMJ^b^	IM + S (by DI); IM + S (by MCS); MCS	FP (1000 Hz) MCS (200 Hz)	MCS and IM + S (by MCS) had lowest CV (2.8%). IM + S (by DI) method had highest CV (3.5%)
Moir et al. [39]	35 male; 35 female	3 CMJ^b^	IM; FT; IM + S (by DI)	FP (1202 Hz)	IM showed the highest intersessions ICC in male participants (0.88–0.96, CV: 1.7–2.8%); in female participants (0.94–0.97, CV: 2.2–3.0%)
Pérez-Castilla et al. [42]	17 male	2 loaded CMJ in each weight (i.e. 17, 30, 45, 60 and 75 kg)	IM; FT	FP (1000 Hz) Smith machine	In free-weight barbell CMJ, IM ((CV = 6.42 ± 2.41%, ICC = 0.88 ± 0.04) and FT (CV = 6.53 ± 2.17%, ICC = 0.88 ± 0.06) In Smith machine CMJ, IM (CV = 11.34 ± 3.73%, ICC = 0.68 ± 0.07) and FT (CV = 5.95 ± 1.12%, ICC = 0.91 ± 0.04)
Dias et al. [15]	20 male; 20 female	15 CMJ^b^	FT; DI	FP (500 Hz) Jump mat (50 Hz) MCS (80 frames.s^−1^)^c^	Not provided
Martínez-Martí et al. [26]	44 male; 17 female	3 CMJ^a^	FT; MCS	Accelerometer (100 Hz) Photocell mat (1000 Hz)^c^ MCS (240 Hz)^c^	Not provided
Buckthorpe et al. [41]	31 males; 9 female	21 CMJ^a^	FT; DI; JAR	Laboratory^c^ and portable FP (2000 Hz) Jump mat (N/A) Belt mat (N/A) Vertical jump device	Not provided
Whitmer et al. [40]	17 male; 18 female	4 CMJ^a^	FT; JAR	FP (1000 Hz)^c^ Jump mat (100 Hz) Vertical jump device	Not provided
García-López et al. [45]	62 male; 27 female	3 CMJ^a^	FT	FP (1000 Hz)^c^ Jump mat (1000 Hz) 2 photocell mats (1000 Hz)	SportJump System Pro photocell mat showed high reliability (CV = 2.98%, ICC = 0.95–0.97) compared with FP (CV = 2.93%, ICC = 0.95–0.97)
Heredia-Jimenez and Orantes-Gonzalez [27]	20 Participants	2 CMJ^b^	FT; IM	FP (200 Hz) with IM method^c^ Photocell mats (1000 Hz) Accelerometer (100 Hz)	Excellent reliability between equipment by using the FT method (ICC = 0.82–0.86) Within the FT method, the photocell mat had higher reliability (ICC = 0.82) than the accelerometer (ICC = 0.74) Using accelerometer with the IM method showed poor reliability (ICC = 0.47)
Słomka et al. [21]	15 male; 16 female	5 CMJ^b^	FT; IM; MCS	FP (1000 Hz)^c^ Photocell mats (1000 Hz) MCS (120 Hz)	The photocell mat showed higher reliability (ICC = 0.98) than MCS (ICC = 0.90) compared with reference, but both are reliable
Bui et al. [30]	23 male; 18 female	2 CMJ^a^	FT; JAR	Jump mat Photocell mat Vertical jump device	Not provided
Leard et al. [29]	25 male; 14 female	2 CMJ^a^	FT; JAR; MCS	Jump mat MCS^c^ Vertical jump device	Not provided
Nuzzo et al. [20]	40 male; 30 female	3 CMJ^a^	FT; JAR	Jump mat Accelerometer (200 Hz) Vertical jump device	The intrasession and intersession ICC was best in accelerometer for male participants (0.95 and 0.88, respectively) and female participants (0.91 and 0.92, respectively)
Aragón [3]	52 male	5 CMJ^b^	FT; IM; IM + S (by MCS); MCS	FP (300 Hz) MCS (60 Hz)^c^	Reliability correlation coefficient for FT method (0.994), IM method (0.986), IM + S method (0.970) and MCS (0.994)
Conceição et al. [32]	14 male; 14 female	3 CMJ^b^	FT; DI; MCS	FP (2000 Hz) Jump mat MCS (200 Hz)^c^ Accelerometer (200 Hz) Self-made Abalakow jump belt	Not provided
Chiu and Dæhlin [1]	29 male; 34 female	3 CMJ^b^	FT; IM; DI; IM + S (by DI); work-energy method	FP (1000 Hz)	Not provided
Wank and Coenning [36]	15 male	4 DJ; 4 CMJ	FT; IM; DI; MCS	FP (2000 Hz) MCS (2 Megapixel resolution)^c^	Not provided
Baca [35]	5 male	DJ from 0.39 m^b^	FT; IM; MCS	FP (1000 Hz) MCS (250 Hz)	Not provided
Brooks et al. [28]	14 male; 12 female	3 CMJ^a^	FT; JAR	FP (400 Hz)^c^ Accelerometer (100 Hz) Vertical jump device Myjump2 Application (via iPad Pro 240 frames. s^−1^ camera)	ICC was 0.91 (90% CI 0.87–0.94) for the accelerometer, and 0.97 (90% CI 0.96–0.98) for the Myjump2 application Intrarater ICC for the Myjump2 application was 0.99

*CMJ* countermovement jumps, *CV* coefficient of variation, *DI* the double integration method, *DJ* drop jumps, *FP* force platforms, *FT* the flight time method, *ICC* intraclass correlation coefficients, *IM* + *S* a positive displacement (i.e. S) is added to the calculated result of the IM method, where the S value can be acquired via either the double integration method or the motion capture systems, *JAR* jump-and-reach method, *M* the impulse-momentum method, *MCS* the motion capture systems

^a^The countermovement jumps with arm swing

^b^The countermovement jumps without arm swing

^c^The reference standard

### Study Characteristics

Of the 21 articles included in the final analysis (see Table 3), one of these studies included the JH measurement during DJ [35], JH during both CMJ and DJ were measured in one study [36], JH during CMJ was evaluated in 19 studies (the CMJ in nine studies were performed without an arm swing [1–3, 5, 15, 21, 27, 32, 39]; the CMJ in eight studies were performed with arm swing [20, 25, 26, 28–30, 40, 41]; the CMJ with and without arm swing were required by authors in one study [24]; participants performed CMJ under loaded condition in one study [42]).

A different number of calculation methods and equipment to derive the outcome measure of JH were utilised in each study. Within the 21 included studies, JH was calculated using different methods (≥ 2) via a single piece of equipment (i.e. the FP) in four studies [1, 5, 39, 42]. The JH-2 calculated by a single calculation method (i.e. the FT method) via different pieces of equipment (≥ 2) was compared in one study [25]. The JH in 16 studies was calculated using various calculation methods (≥ 2) via different pieces of equipment (≥ 2) [2, 3, 15, 20, 21, 24, 26–30, 32, 35, 36, 40, 41]. Further to this, only 12 of the 21 included studies reported the selection of the reference standard or “gold standard” method, and the selections differed between studies. Among these 12 studies, the FP was used as the reference standard (sampling frequency from 200 to 2000 Hz) in six studies [21, 24, 25, 27, 40, 41], while motion capture systems were used as the reference standard in five studies [3, 15, 29, 32, 36], and a photocell mat with motion capture systems as the reference standard in one study [26].

## Discussion

The aim of this systematic review was to critically evaluate the available literature relating to different calculation methods to estimate JH during the CMJ and DJ. When collecting data in applied settings, the equipment and the calculation methods employed may have a significant effect on the outcome measure of JH. Given that a variety of equipment is available to collect FT data, the first sub-section will briefly compare the JH-2 values derived from different pieces of equipment, followed by the explanation of why the FT method over- or under-estimated the JH-2 compared to other calculation methods. The subsequent four subsections critically discuss the advantages and disadvantages of the IM method, the double integration method, the jump-and-reach method and the motion capture systems. Thus, the information in this systemic review makes suggestions for how to standardise procedures and use equipment, and which calculation method to use when assessing JH during the CMJ and DJ tests.

### Flight Time Method

The FT method measures the time intervals between the instant of take-off and landing during vertical jumping (JH-2). This time is then used in the following equation of uniform acceleration, as shown in Eq. 1:

1 $${\text{FT JH-2}} = ut + \frac{1}{2}at^{2} ,$$

where *u* equals the initial velocity that is 0 m/s, *t* is the duration between the take-off and landing instants, where the FT should be half of the *t*, and *a* represents the absolute value of gravitational acceleration (− 9.81 m. s^−2^) [2, 34]. As shown in Table 3, 20 of 21 included studies involved the FT method in their experimental design, mainly because the FT method requires fewer and less complex data calculations and can be used with all equipment discussed here [5, 25].

From the equipment selection perspective, Brooks et al. [28] used a FP with the FT method as the reference standard and reported intraclass correlation coefficients (ICC) of 0.91 (90% confidence interval [CI] 0.87–0.94) for the accelerometer, and 0.97 (90% CI 0.96–0.98) for the My Jump 2 smartphone application. When compared to the FP with the FT method, Heredia-Jimenez and Orantes-Gonzalez [27] reported an ICC of 0.96 when using a photocell mat with the FT method and 0.93 when using an accelerometer with the FT method. These discrepancies in reliability values between studies are likely to be because of the differences in device sampling frequencies, where Brooks et al. [28] set the FP and accelerometer with the sampling frequencies of 400 Hz and 100 Hz, respectively. In contrast, Heredia-Jimenez and Orantes-Gonzalez [27] set their FP and accelerometer with the sampling frequencies of 200 Hz and 100 Hz, respectively, highlighting the importance of higher sampling frequencies for better quality or more reliable data. It is worth noting that, the determination of FT is different between using the FP and accelerometer. When using the FP, FT is identified as the time interval when the vGRF is equal to a force threshold value (e.g. 8 N) [24]. Whereas the accelerometer determines the FT as the time interval when the vertical acceleration is lower or equal to the gravitational acceleration (i.e. − 9.81 m. s^−2^) [43]; thus, establishing why errors appear in the accelerometer [27].

As evidenced by the studies included in this review, the optical measurement systems and jump mats are the most commonly applied equipment for practitioners in the field, but little is known regarding which device offers the strongest reliability [21, 25, 27, 30]. García-López et al. [25] found that compared to the FP with the FT method (0.327 ± 0.056 m), the under-estimation of the JH-2 appeared in both SportJump System Pro (0.314 ± 0.056 m, *p* < 0.05) and ErgoJump Plus (0.269 ± 0.070 m, *p* < 0.001) photocell mats using the FT method. In terms of these two devices, the ErgoJump Plus showed a statistically significantly lower JH-2 compared with the FP along with poor to moderate reliability (coefficient of variation [CV] = 15.94%, ICC = 0.45–0.57). In contrast, the SportJump System Pro photocell mat showed high reliability (CV = 2.98%, ICC = 0.95–0.97) compared to the reference FP (CV = 2.93%, ICC = 0.96–0.97). The under-estimation of the optical measurement systems could be because these systems were placed at a small height off the ground (i.e. 0.7 cm in García-López et al. [25]), where both jump mats and FP were positioned on the ground. At the instant of take-off, the jumpers’ feet are no longer in contact with the ground but still interrupt the transmitter receiver circuit, leading to an under-estimated ascending FT [8]. Whereas the transmitter receiver circuit is interrupted before landing, where the feet have not contacted the ground yet, thus the descending FT is also under-estimated [8]. When using jump mats, the mechanical circuit of the jump mat is triggered by the movement; thus, calculating the time interval between the detection of take-off and landing [25, 44]. If the integrity and hardness are inconsistent across the entire mat surface, the movement that triggers the switch inside the jump mat is likely to be different between different parts of the mat, influencing the measurement of the FT, and thereby the JH-2 [45–47]. Accordingly, the under-estimated FT obtained by the optical measurement systems and jump mats would eventually result in lower JH-2 than estimated by the FP [2, 8, 25]. Researchers have suggested adding the height of the optical measurement devices to the JH-2 measured from these systems when using the FT method, in an attempt to reduce the discrepancy between optical measurement systems and FP or jump mats [25, 30]. In addition, practitioners are advised to consider the body mass of their participants when using jump mats, as it seems likely that additional body mass could trigger the mechanical circuit earlier [45].

Compared to other calculation methods, the FT method has several limitations, which numerous studies have acknowledged [5, 32, 34, 36]. Both the FT and IM methods use the FP to measure the JH-2 from the instant of take-off during jumping, pointing out it is worth comparing these two methods first [5, 36]. To accurately estimate the JH-2, the FT during the ascending and descending phases is presumed equal, which would require the jumper to maintain identical COM positions at the instants of take-off and landing [5, 36]. However, the landing position is lower than the take-off position because of the preparatory ankle dorsiflexion and hip and knee flexion to attenuate landing impact forces [37], making it is hard to achieve a presumed parabolic trajectory of COM position [36, 48]. Thereby, the FT can be artificially extended, which leads to greater JH-2 estimates [32, 34, 36]. To support this, Aragón [3] reported statistically significantly larger JH-2 using the FT method (0.402 ± 0.067 m) than the IM method (0.361 ± 0.066 m, *p* < 0.001). Reeve and Tyler [24] suggested that using the FP with FT method resulted in statistically significantly larger JH-2 compared with the IM method by 2.42 ± 0.31 cm (*p* < 0.001). Supported further by Moir [5], JH-2 calculated by the FT method (male participants: 0.36 ± 0.06 m; female participants: 0.22 ± 0.05 m) showed 3–4% larger values than by the IM method (male participants: 0.35 ± 0.06 m; female participants: 0.21 ± 0.05 m). Therefore, the asymmetric take-off and landing COM positions are the main reason for the difference of JH-2 values calculated by the FT method and IM methods using the FP [36].

The FT method calculates the JH-2 via the time interval from the plantar-flexed take-off to landing on the force–time data, where the take-off height of the jumper is not included in the calculation process. Consequently, this makes the FT method under-estimate JH-2 compared with the double integration method and motion capture systems (i.e., JH-1) [15, 36]. Dias et al. [15] reported that the JH-2 calculated by the FT method (27.59 ± 6.95 cm) was statistically significantly lower than the JH-1 calculated by the double integration method (36.44 ± 7.15 cm, *p* < 0.001) and motion capture systems (37.92 ± 7.46 cm, *p* < 0.001). In addition, a statistically significantly lower JH-2 was measured by the FT method using the jump mat (38.6 ± 6.5 cm) compared to the JH-1 measured by the double integration method using the FP (50.3 ± 7.5 cm, *p* < 0.05) in the study by Buckthorpe et al. [41]. Research from Wank and Coenning [36] also showed statistically significantly lower JH-2 estimated from the FT method than the JH-1 from the motion capture systems in CMJ (*p* < 0.001) and DJ (*p* < 0.001). Thus, the rise in height generated by plantarflexion of the ankles prior to the take-off instant largely explains the higher JH-1 values calculated by the double integration method and motion capture systems [15, 36, 41]. However, this explanation is not in agreement with other studies, where Leard et al. [29] revealed no statistically significant differences between JH estimated by the FT method using jump mats (44.17 ± 10.29 cm) and motion capture systems (43.79 ± 10.29 cm, *p* = 0.972). Noting that Leard et al. [29] did not make reference to how they define the JH and nowhere in their methods section was it clarified that they calculated JH-1 or JH-2 via different methods. The most likely interpretation could be that they measured the COM displacement from the instant of take-off to landing during CMJ via different methods. Thus, the JH-2 values calculated by Leard et al. [29] may not be significantly different between the FT method and the motion capture systems. In addition, both Martínez-Martí et al. [26] and Słomka et al. [21] used the position-time data at the take-off and landing to determine the FT, then calculating the JH-2 via the equation of uniform acceleration (i.e. Eq. 1). Thereby, the JH-2 calculated by the FT method in their studies showed no statistically significant differences from the motion capture systems (*p* > 0.001 and *p* > 0.05, respectively). Thus, the FT method provides similar outcomes to the motion capture systems, but only if measuring JH-2 where the take-off height is not considered [3, 21, 26]. One thing that should be noted is Martínez-Martí et al. [26] required participants to keep their lower extremities fully extended during the instant of take-off and landing, whilst Słomka et al. [21] recruited professional volleyball athletes who are likely to have excellent and consistent jump technique. Cumulatively, these requirements might, to some extent, maximise the symmetric COM position during take-off and landing, thereby minimising the discrepancy between the FT method and other calculation methods.

Although the accuracy of the FT method is primarily determined by the aforementioned factors, this method is still suitable for various sports testing environments because of its simple operation, fewer data processing and abundant equipment available (e.g. optical measurement systems, jump mat, FP and smartphone applications) [21]. If the FT method is selected as the calculation method, some corrective equations proposed by Bui et al. [30] or Wade et al. [2] could be used to eliminate factors such as the take-off and landing positions or foot size that might influence the accuracy of subsequent data. In addition, given that there may be 1–2 cm differences between methods and equipment when measuring JH, practitioners are suggested to ensure the equipment, methods and requirements are consistent between test sessions [2, 21].

### Impulse-Momentum Method

The IM method is based on Newtonian mechanics and related mechanical laws. Specifically, the IM relation is derived from Newton’s law of acceleration, which is also connected to the law of conservation of energy [5]. Accordingly, the potential energy at the maximum height during the flight phase is identical to the kinetic energy of the jumper at take-off [34, 36]. The net vertical force is calculated from the vGRF reading from the FP minus the jumper’s body weight. This net vertical force is then numerically integrated, typically using the trapezoid rule, from the start of the propulsion phase to the instant of take-off [5, 36]. Finally, the net impulse obtained via integration of the net vGRF is equal to the vertical momentum of the jumper, which is the product of body mass and the velocity at take-off [31]. This process is shown in Eq. 2:

2 $$J = \mathop \int \limits_{{t_{{{\text{start}}}} }}^{{t_{{\text{take - off}}} }} \left( {F_{{{\text{vGRF}}}} - F_{{\text{g}}} } \right)dt = m v_{{\text{take - off}}} - m v_{{{\text{start}}}} ,$$

where *J* is the net impulse, and *t*_start_ and *t*_take-off_ are the time at instant of the propulsion phase and take-off, respectively. The *v*_start_ (*v* = 0) and *v*_take-off_ are the velocity at *t*_start_ and *t*_take-off_, respectively. The *F*_vGRF_ and *F*_g_ are the vGRF and the body mass of the participant, respectively. Finally, the *v*_take-off_ is extracted from Eq. 2 by dividing the net impulse by the body mass, which the *v*_take-off_ is subsequently used for the calculation of JH-2 via Eq. 3:

3 $${\text{IM JH-2}} = { }\frac{{\left( {v_{{\text{take - off}}} } \right)^{2} }}{2g},$$

where *g* represents the acceleration of gravity (− 9.81 m. s^−2^).

As previously mentioned, the accelerometer provides reliable but inaccurate JH-2 compared to the FP using the FT method [27]. Not surprisingly, the JH-2 measured by the accelerometer was statistically significantly higher than the FP using the IM method by 0.07 m (*p* < 0.001), along with the accelerometer showing poor reliability (ICC = 0.47) [27]. Although both accelerometer and FP calculate the JH-2 using the velocity at take-off via Eq. 3, factors like the placement of the accelerometer device and the trunk rotation with respect to the coronal and sagittal axes inaccurately quantify the velocity of moving COM [20, 27, 43, 49]. Therefore, using the IM method via the FP provides a more accurate and reliable JH-2 estimation than an accelerometer [27].

From the calculation method perspective, an early study by Moir et al. [39] confirmed that both FT and IM methods were highly reliable (CV < 2.9%, ICC > 0.87) when measuring JH-2. Because of the FT method often over-estimating JH-2 values, Słomka et al. [21] reported higher but not statistically significant JH-2 values using the FT method compared to the IM method (*p* > 0.05), and both methods presented excellent reliability (FT: CV = 0.10%, ICC = 0.92; IM: CV = 0.11%, ICC = 0.91). To investigate which method is suitable to evaluate the loaded CMJ, Pérez-Castilla et al. [42] recruited 17 male participants and analysed their JH-2 during loaded CMJ (load range: 17 kg, 30 kg, 45 kg, 60 kg and 75 kg) performed in a Smith machine and with free-weight barbells. In accordance with previous studies, they revealed that the reliability of JH-2 was comparable between the IM method (CV = 6.42 ± 2.41%, ICC = 0.88 ± 0.04) and the FT method (CV = 6.53 ± 2.17%, ICC = 0.88 ± 0.06) during the free-weight barbell-loaded CMJ; but it was better for the FT method (CV = 5.95 ± 1.12%, ICC = 0.91 ± 0.04) when the loaded CMJ was performed in a Smith machine (CV = 11.34 ± 3.73%, ICC = 0.68 ± 0.07 for the IM method) [42]. Results showed both methods were reliable to evaluate the loaded CMJ, but the relative lower reliability in the IM method suggested that when measuring the JH-2 with the Smith machine, the friction force with the linear bearings of the Smith machine reduces the accuracy of the IM method [42]. Although both the FT and IM methods derive the JH-2 via the equations of uniform acceleration, the JH-2 estimated by the FT method is affected by the change of COM positions upon take-off and landing, where the change in COM positions is likely to generate variations in the FT [5, 39, 50]. In contrast, the IM method calculates the JH-2 via the take-off velocity, which depends upon the net vertical impulse (i.e. positive vertical impulse minus negative vertical impulse) and jumpers’ body mass, where the IM method is unaffected by the asymmetric take-off and landing COM positions [39, 51]. Moir et al. [39] found that although the positive (CV = 1.7–5.5%, ICC = 0.89–0.98) and negative vertical impulses (CV = 4.0–8.8%, ICC = 0.82–0.96) presented large variations, the take-off velocity was very reliable irrespective of sexes (CV = 1.7–3.2%, ICC = 0.87–0.97). The compensatory strategies within the motor system produce the reciprocal alterations in positive and negative vertical impulses, thereby ensuring that the measured outcomes (i.e. JH-2 values) between trials are preserved [39]. Thus, in accordance with previous investigations [5, 24, 39], the IM method calculates more accurate and reliable JH-2 values compared to the FT method, when both methods are calculated from FP.

Nevertheless, like the FT method, the IM method calculates the JH between the COM position at the take-off and the apex of the jump (i.e. JH-2), and only accounts for a fraction of the work performed during the jump [3, 5, 15]. For example, Wank and Coenning [36] measured CMJ and DJ performance via the FP, and reported that in both jump actions, the IM method calculated statistically significantly lower JH-2 than the motion capture systems (JH-1, *p* < 0.01) and double integration method (JH-1, *p* < 0.01). Similarly, the JH-2 measured by the IM method (29.8 ± 8.9 cm) was found to be statistically significantly lower than the JH-1 measured by the double integration method (42.0 ± 9.4 cm, *p* = 0.517) in a study by Chiu and Dæhlin [1]. Their findings highlighted that the IM method fails to measure the work done by the plantarflexion of the ankles to evaluate the COM vertically before the take-off, which explains why lower JH-2 values are estimated by the IM method. Although it was shown that the IM method removes many of the confounding variables when using the FT method (e.g. take-off and landing COM positions) [5], there are still concerns regarding using the IM method for the JH-2 measurement. First, compared to the FT method, the IM method involves the numerical integration, which potentially generates some calculation errors [1, 2, 36], and requires accurate body mass estimation and data treatment (i.e. filtering) [33]. Second, the accuracy of the IM method depends on the precise selection of the instant of take-off, which means the “meaningful change in force” on the force–time curve should be accurately selected [42, 52]. Otherwise, misidentifying the instant of take-off by just 2–3 ms can result in a difference of about 2% in velocity where this imprecise velocity value can further affect calculation of JH-2 via Eq. 3 [18, 48]. Whereas only some of the included studies defined the take-off instant as the vGRF being equal to 0 N [2, 21, 36], less than 8 N [24], less than 10 N [42] or less than the peak residual (i.e. peak difference between vGRF and 0 N) during flight [5, 39]. Therefore, future studies could consider defining the take-off instant as ± five times the vGRF measured over a 0.3-s period during the flight phase where the participants are no longer in contact with the ground [53]. The 0.3 s was chosen because participants are likely to produce the FT greater than 0.3 s [5, 53]. This method might, to some extent, best represent the instant of take-off and minimise the influence of noise from the FP [54]. Chavda et al. [54] in addition suggested to use the vGRF extracted from only the middle part of the flight phase instead of over a 0.3-s period. This alternative approach would also help to evaluate jumpers who cannot generate the FT longer than 0.3 s (e.g. loaded jump conditions, participants with insufficient jump technique) [10, 54].

Furthermore, it would be possible to obtain the displacement–time data by twice integrating the force–time data from initial standing still to landing [41], and then calculating the COM displacement (JH-2 value) from the COM height at take-off to the apex of the flight phase. However, twice integration processes would accumulate more calculation errors, making the calculated JH-2 values inaccurate compared with the IM method [55]. Based upon the comparisons of this systematic review, when the FP is available for the data collection, practitioners are encouraged to calculate the JH-2 (i.e. the COM displacement before the take-off is ignored) using the IM method [5].

### Double Integration Method

Given that the FT method calculated JH-2 according to the time intervals from take-off to landing [5, 34], the IM method integrates the vGRF from the initiation of the propulsion phase to take-off, in which the COM take-off height is unknown in both methods [5]. The double integration method integrates the force–time data twice from the movement initiation to the landing instant to obtain an entire displacement–time curve during jump actions [32, 36]. The COM displacement trajectory at its highest point is considered the JH, as shown in Eq. 4,

4 $$~{\text{DI JH-1}} = \int {\int_{{t_{{{\text{landing}}}} }}^{{t_{{{\text{start}}}} }} {\left( {F_{{{\text{vGRF}}}} - F_{{\text{g}}} } \right){\text{d}}t~ + ~h_{0} ,} }$$

where *t*_start_ and* t*_landing_ are the time at instant of countermovement (or drop movement in DJ) and landing, respectively. The *F*_vGRF_ and *F*_g_ are the vGRF and the body mass of the participant, respectively. The *h*_0_ in CMJ is the COM height of jumpers during initial standing still (i.e. *h*_0_ = 0 m), and the *h*_0_ in DJ is the drop height. It is worth noticing that the DJ measures via the above equation are applicable only when the two-adjacent FP are available [56].

From the calculation method perspective, previous studies like Conceição et al. [32], Wank and Coenning [36] and Wade et al. [2] have found that the double integration method is one of the most reliable and accurate approaches to evaluate the JH-1 when using the vGRF. In addition, all aforementioned studies agreed that only the double integration method via FP could measure the JH-1 with the most negligible difference from the motion capture systems [2, 32, 36]. In contrast with the previous three studies [2, 32, 36], Dias et al. [15] reported that the JH-1 measured by the double integration method (36.44 ± 7.15 cm) was statistically significantly different from the motion capture systems (37.92 ± 7.46 cm, *p* < 0.01). Like the IM method, the double integration also relies on the reading of vGRF from the FP and involves the numerical integration process [1, 25], where the sampling frequency of the FP might somewhat influence the JH-1 measurement [1]. When FP was set at 2000 Hz, Conceição et al. [32] and Wank and Coenning [36] revealed that there was no statistically significant difference between the JH-1 measured by the double integration method and motion capture systems (*p* = 0.079 and *p* > 0.01, respectively). Similarly using the FP with 1000 Hz, JH-1 was not statistically significantly different between the double integration method (0.432 ± 0.15 m) and the motion capture systems (0.429 ± 0.12 m, *p* > 0.05) [2]. However, when the sampling frequency dropped to 500 Hz, a statistically significant difference between the double integration method and motion capture systems (*p* < 0.01) was observed [15]. Therefore, it could be hypothesised that considering the motion capture systems as the reference standard, the double integration method is accurate when the sampling frequency of the FP is equal to or larger than 1000 Hz. Conceição et al. [32] explained that when using the FP with a lower sampling frequency (i.e. < 1000 Hz), the recorded force–time data are likely to include some fluctuations or undefined events during the quiet standing period and flight phase, which eventually influences the estimation of body mass or movement initiation, thereby affecting the JH-1. However, limited studies are included in this systematic review (*n* = 21), and authors in only four studies measured the JH-1 values using the double integration method concurrently with the motion capture systems [2, 15, 32, 36]. It would be recommended that future studies use the FP with various sampling frequencies (e.g. 500 Hz, 1000 Hz, 1202 Hz and 2000 Hz) to measure JH (i.e. including both JH-1 and JH-2). These JH values are then compared to the reference motion capture systems to investigate whether the level of sampling frequency influences the accuracy and reliability of the double integration method [2].

The double integration method is considered reliable during CMJ measures because this method starts the twice integration prior to the movement initiation of CMJ (i.e. standing with flat feet), where the initial standing height is a constant value, and a ‘truly’ zero acceleration is achieved, which is the requirement for accurate integrations [1, 2, 32, 36]. It is important to remember that the ankle plantarflexion before take-off makes the COM move upwards or generates a positive vertical displacement, in which the COM height at take-off is higher than standing still [1, 3, 57]. As mentioned above, neither the FT method nor IM method takes the COM height at take-off into account in their calculation of JH-2 [1–3, 41, 48, 57]. In order to eliminate the discrepancy between the IM and double integration methods, several studies were in line with applying twice integration to the force–time curve (from the movement initiation to the take-off instant) to obtain the positive displacement (i.e., S) generated by the ankle plantarflexion before the take-off, then adding this ‘S’ to the IM method calculated JH-2, i.e. IM + S method [1, 3, 5, 57]. Moir [5] reported a high degree of consistency across methods in male participants (ICC = 0.927, 95% CI 0.887–0.955) and female participants (ICC = 0.934, 95% CI 0.897–0.960). They also found that the IM + S method measured JH-1 with lower variability (male participants: CV = 12.0%; female participants: CV = 15.3%) compared to the IM method (male participants: CV = 16.2%; female participants: CV = 22.2%). Chiu and Dæhlin [1] observed a perfect agreement between the double integration and IM + S methods (42.0 ± 9.4 cm and 42.0 ± 9.4 cm, *p* = 1.000) when measuring JH-1 via FP. Further to this, no statistically significantly different JH-1 between IM + S method and the motion capture systems (43.20 and 42.90 cm, *p* > 0.05) was found by Wade et al. [2]. Despite these results highlighting a possible solution to reduce the discrepancy of calculated JH between the IM method, double integration method and the motion capture systems, more studies would be required to investigate whether the IM + S method can provide practitioners valid, reliable and accurate JH (i.e. including both JH-1 and JH-2). It is worth noting that the calculated positive displacement generated by the ankle plantarflexion prior to take-off is influenced by some non-modifiable factors, such as foot length, where a longer foot length is likely to evaluate the COM height more when the ankle plantarflexion angle is the same [1].

As proposed by Baca [35], the double integration process could be applied in the backward sequence via a single FP if two-adjacent FP are unavailable during the DJ evaluation. In addition, Costley et al. [12] mentioned that the drop height is an essential parameter that determines the accuracy of measurement during the DJ. In this instance, the COM height (*h*_0_) equals zero as the jumpers have landed, so applying the integration process in reverse makes the calculation of drop height in the forward integration process unnecessary [35, 36]. Noticing that the backward integration requires the jumpers to stand still and remain in a rigidly upright position afterwards landing for at least 1 s, which might challenge jumpers’ maintenance of balance as the surface area of a single FP is much smaller than two-adjacent FP [36, 56, 58, 59]. Although the double integration method has been used in previous studies [2, 15, 32, 36], twice integrating the data accumulates measurement errors and more linearity [2, 55], and this method is very sensitive to the accurate determination of jumpers’ body mass [10, 55]. However, compared with the motion capture systems that require extensive equipment preparation and later data analysis, the double integration method using the vGRF data recorded by a portable FP is more practical for those working in the field [15]. Thus, in agreement with previous investigations [15, 32, 36], practitioners are encouraged to quantify the COM displacement between the COM height at the initial standing and apex of the jump (i.e. JH-1) using the double integration method (via the FP).

### Jump-and-Reach Method

The jump-and-reach method via the vertical jump devices has been proposed to make the JH measurement more convenient for various tests in the field because the method needs less equipment and provides the outcome directly [30]. Practitioners commonly use the Vertec vanes or the Sargent jump [40]. The Sargent jump is performed by jumpers who have tape or chalk on their fingers, who then jump and slap the fingers against a wall [40]. Subsequently, the difference between the standing touch height and jumping touch height is defined as the JH-1. Similarly, the Vertec vanes device consists of several plastic swivel vanes (i.e. separated by half-inch [or 1.27-cm] increments) mounted on a telescopic metal pole that can be adjusted to the jumpers’ standing reach height, while jumpers were told to jump and displace the highest vane they can. The JH-1 is then estimated by subtracting the height of the highest vane touched during flight from the height of the vane touched during quiet standing [40].

When comparing the difference in JH between methods, authors in six studies adopted the jump-and-reach method, and existing results again appeared to be somewhat inconsistent. Bui et al. [30], Brooks et al. [28] and Buckthorpe et al. [41] agreed that the JH-2 values measured by the FT method were statistically significantly larger than the JH-1 values estimated from the jump-and-reach method by at least 5 cm (*p* < 0.05, *p* < 0.05 and *p* < 0.001, respectively). Given that the Vertec device is calibrated using flat feet standing on the floor, the jump-and-reach method (which measures JH-1) involves the positive vertical COM displacement generated by the ankle plantarflexion prior to take-off [40]. In contrast, the FT method does not detect this displacement, which partially explains why the over-estimation appears in the jump-and-reach method [10, 28]. In order to test whether the jump-and-reach method is reliable compared to the FT method, it is suggested to measure the standing reach height at an ankle plantarflexion situation instead of flat feet standing [28, 40, 60]. This modification fixes the contrast variable at the JH-2 values and eliminates the effects of COM displacement before take-off; thus, providing a fairer comparison between the FT method and the jump-and-reach method [60]. In contrast, not all studies have agreed that the jump-and-reach method always over-estimates JH. Nuzzo et al. [20] required participants to touch the Vertec device with both hands. The maximum JH-2 in their study was statistically significantly higher measured by the jump mat using the FT method (male participants: 57.25 ± 9.0 cm; female participants: 38.25 ± 6.0 cm) than the JH-1 measured by the jump-and-reach method (male participants: 49.78 ± 9.1 cm; female participants: 31.65 ± 5.9 cm, *p* < 0.05). Furthermore, the intersession reliability measures in this study indicated that in female participants, the jump-and-reach method (CV = 8.6%, ICC = 0.80) was less reliable as opposed to the FT method (CV = 4.4%, ICC = 0.92); in male participants, a higher intersession reliability was found with the jump-and-reach method (CV = 5.9%, ICC = 0.90) rather than the FT method (CV = 6.3%, ICC = 0.84) [20]. Of note as well, jumpers in this study were also required to keep their heads and eyes level, and they could not look at the Vertec vanes. These requirements might, to some extent, compromise the coordination of the arm swing and prevent jumpers from displacing the vanes at the peak height of their jumps, resulting in lower JH-1 values [20]. Although similar results were given by Leard et al. [29], a lack of JH definitions makes it challenging to interpret their findings. In study by Whitmer et al. [40], they did not reveal statistically significantly different JH between the jump-and-reach method (JH-1: 0.48 ± 0.10 m) and the FT method using the jump mat (JH-2: 0.50 ± 0.12 m, *p* > 0.01). Whitmer et al. [40] estimated the FT (via the jump mat) using proprietary algorithms instead of the simple projectile motion equation (i.e. Eq. 1). This algorithm added approximately 100 ms of time to the FT measured by the jump mat, thereby achieving this closer comparison between the two methods. Authors in the same study also estimated the FT using the FP (0.524 ± 0.078 s) and found a statistically significantly lower FT compared with the jump mat (0.629 ± 0.077 s, *p* ≤ 0.01) [40]. However, a statistical comparison was missing between JH-2 calculated by the lower FT that comes about from the FP (via Eq. 1) and JH-1 from the jump-and-reach method. Thus, whether their result is consistent with previous studies that suggest the over-estimation appears in the jump-and-reach method is unknown [28, 30, 41].

Despite the appeal of the jump-and-reach method, factors that influence the accuracy of the jump-and-reach method should not be ignored. First, the accuracy depends on the timing of the touch, which is the ability of jumpers to displace the vane or touch the wall at the peak height of jumping. If touching of the device does not appear during the peak height, the measured JH-1 via the jump-and-reach method will be under-estimated [20, 29]. Second, in order to touch the device at the peak height, jumpers are required to have good coordination of arm swing and jump, which means jumpers who previously experienced jump training (e.g. volleyball spiking, basketball rebounding) or associated with better skills on the jump-and-reach test are likely to reach higher [20]. In comparison, those participants without any jump test experience may need multiple familiarisation trials prior to the data collection, to ensure these participants provide a valid JH-1 [20, 29]. Third, the insufficient range of arm flexion may prevent jumpers from touching at the highest point, thereby resulting in an under-estimated JH-1 [20]. Fourth, the sensitivity of the Vertec device also influences its accuracy because the space between each vane makes this device only measure the JH-1 in the 1.27-cm increments [20, 31]. In this instance, if jumpers touch the space between two vanes, the measured JH-1 is mistakenly shown by the highest vane displaced rather than the actual touch point between two vanes. Therefore, this potential error explains why the over-estimation of JH-1 appears in the study as mentioned earlier [28, 30, 41].

In addition, it is not surprising to see the JH difference between the jump-and-reach method and other calculation methods (e.g. the FT method), as they measured disparate biomechanical constructs, i.e. the reaching height difference versus the FT, which the latter variable is associated with the jumpers’ COM displacement [20]. Consequently, the jump-and-reach method is recommended if practitioners would like to know the maximal jump-and-reaching height, which is a specific test parameter in volleyball and basketball [20, 61]. Otherwise, if practitioners are interested in quantifying the maximal vertical COM displacement from the initial standing to the apex during jumping (i.e. JH-1), the double integration method via the FP is preferred [15, 32, 36]. Alternatively, if the interest is to estimate the maximal vertical COM displacement from the take-off instant to the apex during jumping (i.e. JH-2), the IM method via the FP is recommended [5, 39].

### Motion Capture System

The motion capture systems typically involve high-speed cameras or multiple 3D cameras. The 3D motion capture system acquires the position-time data by tracking the reflective markers placed on the trunk, pelvic and lower extremities [2], the left and right femoral condyles [32], or the total body bony landmarks (i.e. 47 makers) [36]. Subsequently, a mathematical body model reflects the COM position is built, and the JH-1 is estimated by quantifying the peak COM height of the model during the flight relative to the initial height taken while the participant is standing still [2].

Compared to the double integration methods via the FP, the motion capture systems eliminate issues of integration errors, making the calculated JH-1 closer to the real value, which allows it to be widely used as a reference standard [2, 32, 36]. As aforementioned, Wank and Coenning [36] revealed slightly higher but not statistically significant JH-1 from the double integration method via the FP than the motion capture systems (*p* > 0.01). Similar results have been noted in Conceição et al. [32], the double integration method via the FP only over-estimated the JH-1 by 0.15 ± 0.13 cm in contrast to the motion capture systems (*p* = 0.079). Although the difference is relatively minor, factors that affect the COM estimation and accuracy of the motion capture systems should be highlighted. For example, researchers in some studies only model parts of the total body for the COM estimation (e.g. pelvic kinematic method [62] or two markers on the femoral condyles [32]), which these models’ COM are somewhat different from the body’s COM estimated by the FP, as the FP measures the vGRF acting at the true body’s COM [62]. Of note as well, markers attached to the pelvic area are influenced by the tilt or rotation of the pelvic during flight [32], markers attached to the lower limbs are affected by the lower limb extension when taking off [2], while the arm swing could raise the COM height at the take-off instant, which may not be detected by pelvic markers [1]. Further to this, markers shifting relative to the bony landmarks [2, 36], the inadequacy of the mathematical body model, software that used to build the mathematical body model and a lower sampling rate (< 250 Hz) [35] can accumulate errors when using the motion capture systems.

In addition, when evaluating the DJ with a high-speed camera placed in front of the jumpers, the accuracy of JH measures is influenced by an improper drop technique [35]. In short, if the drop action has started, but the foot is still in contact with the drop platform, the front placed camera tends to under-estimate the vertical COM position, leading to an inaccurate drop height and rebound JH [35]. To cover the deficit that using the motion capture systems alone may not accurately detect the movement initiation, Baca [35] suggested using the motion capture systems concurrently with the FP to enhance the reliability of JH measurement during CMJ and DJ. Specifically, the key timepoints (e.g. the movement initiation, touchdown and take-off) in jump actions are identified first on the force–time curve. These timepoints are then tracked back to find their vertical coordinates on the position-time data, for the subsequent calculation of JH [35].

Compared to the double integration method that requires the FP, estimating the JH-1 via the motion capture systems is not recommended, given that the system involves numerous errors during COM estimation and requires rather extensive set-up processes [3, 5, 15]. Interestingly, Conceição et al. [32] pointed out that the FP needs a reaction time to let the measured vGRF decrease to 0 N. Thereby, in their study, the FT estimated from the velocity–time data via the FP (i.e. the period between the maximum and minimum velocity) showed lower values than the FT estimated from the motion capture systems (i.e. the period between the position data is zero) [32]. Noting that although the FP and motion capture systems are able to measure the same parameters simultaneously, the outcomes might somewhat differ.

### Limitations and Suggestions for Future Research

Some limitations of this systematic review must be outlined. First, only two studies examining the JH calculation during the DJ were included in this review. The limited number of DJ studies makes it insufficient to provide any definitive conclusions regarding which method or equipment is best to determine JH during this test. Thus, more studies are needed to quantify JH in the DJ using different pieces of equipment and calculation methods. Second, no studies utilizing linear position transducers met the inclusion criteria for the review. Therefore, it is difficult to say whether this device should be recommended for practitioners, when aiming to quantify JH. Future research is encouraged to use different devices to investigate the reliability of JH calculation methods during CMJ and DJ.

Given that different pieces of equipment are likely to have different amounts of error, future studies should consider several factors that can generate discrepancies when comparing JH values measured from the FP and motion capture systems. First, the FP and motion capture systems need appropriate sampling frequencies to synchronise the force–time and position-time data (e.g. 2000 Hz and 250 Hz, respectively) [35, 63], while the sampling frequency of the FP should be higher than 1000 Hz if integration of force data is required [32]. Second, it is of great importance to clearly define the JH (i.e. JH-1 or JH-2) whilst ensuring the JH values being compared between two devices are equal [2, 3]. Further, given the inherent differences in how JH-1 and JH-2 are computed, a comparison would be meaningless if the JH-2 (derived from the IM method via the FP) and the JH-1 (derived from the motion capture system) were directly compared [3]. Finally, the identification of key timepoints (e.g. take-off instant and landing) should be consistent between devices, indicating an equal threshold should be used to define these key timepoints during jumping [32].

## Conclusions

**Fig. 2:**
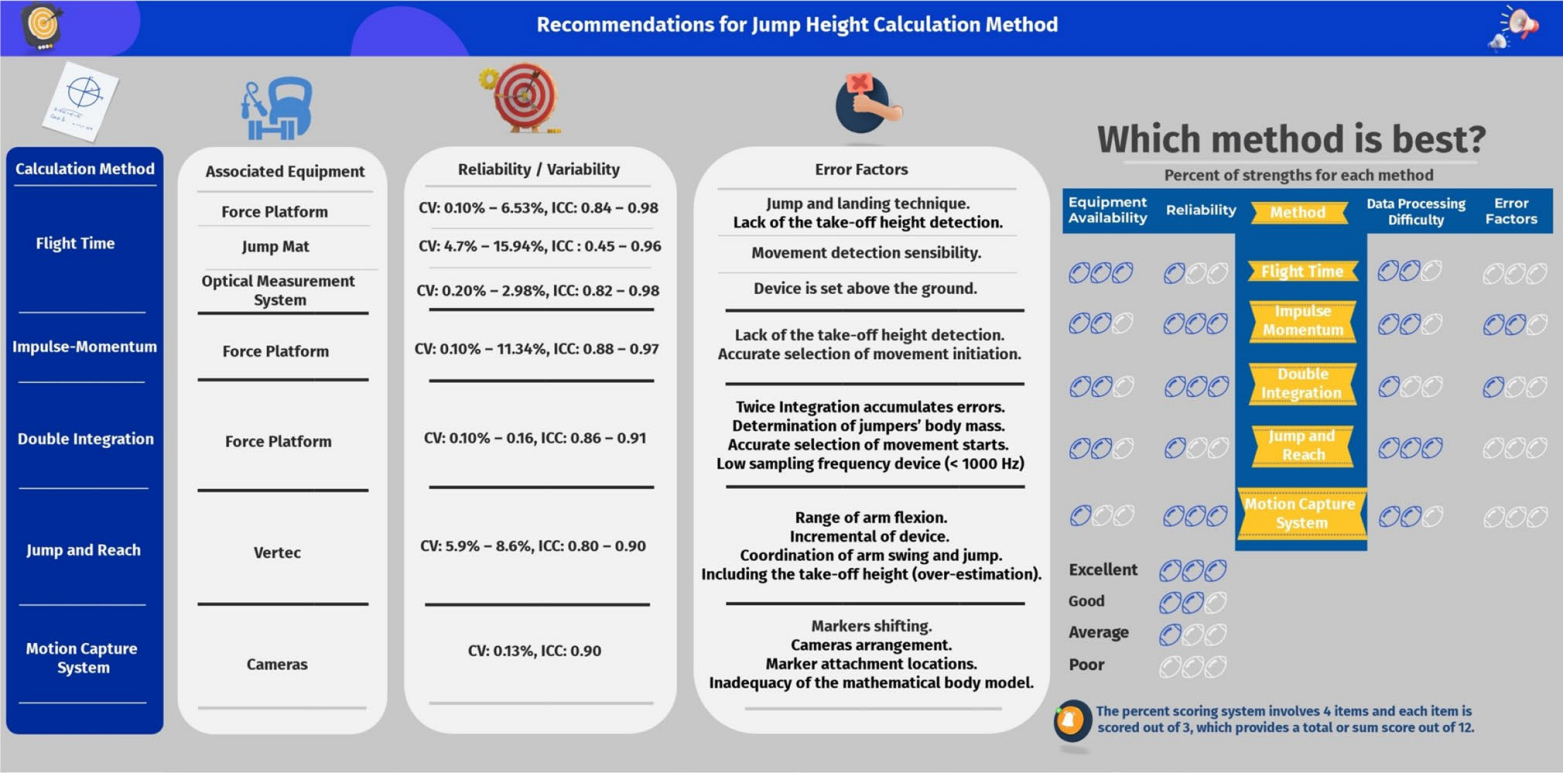
Recommendations for jump height calculation methods (courtesy of www.Visme.co). *CV* coefficient of variation, *ICC* intraclass correlation coefficient

The cumulative body of literature indicates that the measured JH is influenced by the calculation methods and equipment employed. For measuring the JH from the COM height at the initial flat feet standing to the apex of jumping (i.e. JH-1), the double integration method via the FP is encouraged for practitioners, as this method measures the most comparable JH-1 values compared to the motion capture systems. Of note as well, when two-adjacent FP are unavailable in the DJ measurement, the double integration method is unable to calculate the initial standing height, and the integration process must be processed reversely. The motion capture systems are not preferred, given that this method requires accurate COM estimations and is primarily determined by the equipment availability. For measuring the JH from the COM height at the instant of take-off to the apex of jumping (i.e. JH-2), we recommended that practitioners use the IM method via the FP when estimating JH-2 values in both CMJ and DJ, where the COM displacement before the take-off is ignored. The IM method requires a simpler integration process than the double integration method and shows excellent reliability. The FT method may be of use because of its simple calculative process and an abundant equipment selection enables practitioners to conduct the test when working with large groups of athletes. However, some factors such as the take-off and landing positions reduce the accuracy of the FT method, and practitioners should be aware of this. Similarly, the jump-and-reach method is the most convenient approach to estimate the JH-1 and the maximum jump-and-reach height when testing jumpers in a big squad, despite this method showing lower reliability in some studies. Therefore, if the jump-and-reach method is the only option to quantify JH-1, practitioners are suggested to minimise factors such as the coordination of the jump and arm swing or the timing of touch that affects the accuracy of the jump-and-reach method before conducting the data collection. The findings of this systematic review emphasise the strengths and weaknesses of each calculation method during the calculation of JH in CMJ and DJ (as shown in both Table 4 and our complementary infographic, Fig. 2). Our findings highlight the requirement for further investigation regarding the reliability of each calculation method under different equipment settings.

**Table 4:** Recommendations for jump height calculation methods

Calculation method	Associated equipment	Reliability/variability	Error factors
Flight time	Force platform	CV: 0.10–6.53%, ICC: 0.84–0.98	Jump and landing technique Lack of the take-off height detection
	Jump mat	CV: 4.7–15.94%, ICC: 0.45–0.96	Movement detection sensibility
	Optical measurement system	CV: 0.20–2.98%, ICC: 0.82–0.98	Device is set above the ground
Impulse-momentum	Force platform	CV: 0.10–11.34%, ICC: 0.88–0.97	Lack of the take-off height detection Accurate selection of take-off instant
Double integration	Force platform	CV: 0.10–0.16%, ICC: 0.86–0.91	Twice integration accumulates errors Determination of jumpers’ body mass Accurate selection of movement starts Low sampling frequency device (≤ 1000 Hz)
Jump and reach	Vertec	CV: 5.9–8.6%, ICC: 0.80–0.90	Range of arm flexion Incremental of device Coordination of arm swing and jump Including the take-off height (over-estimation)
Motion capture system	Cameras	CV: 0.13%, ICC: 0.90	Markers shifting Camera arrangement Marker attachment locations Inadequacy of the mathematical body model

*CV* coefficient of variation, *ICC* intraclass correlation coefficient

## Declarations

### Availability of Data and Material

The data within this systematic review are secondary data and available through the relevant articles referenced throughout. Data are available upon request.
